# Comparison of Total Antioxidant Capacity of Saliva in Women with Gestational diabetes mellitus and Non-diabetic Pregnant Women

**DOI:** 10.4317/jced.53845

**Published:** 2017-11-01

**Authors:** Ulduz Zamani-Ahari, Sahar Zamani-Ahari, Zahra Fardi-Azar, Parisa Falsafi, Milad Ghanizadeh

**Affiliations:** 1 Assistant professor, Department of Oral Medicine, Faculty of Dentistry, Ardabil University of Medical Sciences, Ardabil, Iran; 2 PHD Student, Department of Food Hygiene, Faculty of Veterinary Medicine, Ferdowsi University of Mashhad, Mashhad, Iran; 3 Professor, Department of Obstetrics and Gynecology, Alzahra Hospital, Tabriz University of Medical Sciences, Tabriz, Iran; 4 Assistant professor, Department of Oral Medicine, Faculty of Dentistry, Tabriz University of Medical Sciences, Tabriz, Iran; 5 Post-graduate Student, Department of oral and maxillofacial surgery, Faculty of Dentistry, Tabriz University of Medical Sciences, Tabriz, Iran

## Abstract

**Background:**

Pregnancy is considered a stressful event, results in higher levels of oxidative stress and considerable changes in physiological and metabolic functions such as gestational diabetes mellitus (GDM). Due to the cumulative effect of antioxidants and considering the controversies in this area, this study was undertaken to investigate the total antioxidant capacity (TAC) of saliva in pregnant women whit gestational diabetes in comparison to non-diabetic pregnant women.

**Material and Methods:**

In this cross-sectional study (2015-16), a total of 31 women with a diagnosis of GDM and 59 non-diabetic pregnant women were included in the diabetic and control groups, respectively. Salivary samples were collected by spitting method. When all samples were collected, total antioxidant capacity (TAC) was measured with the use of a commercial kit following the manufacturer’s instructions. Data were analyzed with descriptive statistics and Mann-Whitney test using SPSS 18.

**Results:**

Average TAC level in the saliva of women with gestational diabetes was 0.10 ± 0.14, with 0.04 ± 0.11 in non-diabetic pregnant women. Nonparametric Mann-Whitney test showed that this difference was statistically significant (*P*=0.024).

**Conclusions:**

Under the limitations of the present study it can be concluded that there is an increase in oxidative stresses during pregnancy, followed by an increase in the total levels of salivary antioxidants to counteract such stresses. Therefore, it is expected that determining the salivary antioxidant levels during pregnancy can be an alternative technique for the early diagnosis of diabetes.

** Key words:**Gestational diabetes mellitus, pregnancy, saliva, total antioxidant capacity.

## Introduction

Gestational diabetes mellitus (GDM) is abnormal glucose metabolism during pregnancy (1,2), with a prevalence rate of 1.7-11.6%(3). GDM patients might be affected by pregnancy induced hypertension, polyhydramnios, infections, ketoacidosis and other conditions. Long-term poor control of blood glucose levels might give rise to chronic fetal hypoxia, disturbances in fetal growth, malformations, neonatal hyperbilirubinemia, hypoglycemia, respiratory distress syndrome, etc (1,4,5).

The exact etiology of GDM is still to be elucidated. Clarification of pathogenesis is important for early diagnosis and treatment, which improve the maternal and infant prognoses. Recently, researchers have focused their attention on the relationship between oxidative stress and GDM (3,6,7).

Pregnancy is considered a stressful event, resulting in considerable changes in physiological and metabolic functions (6,8). In addition, pregnancy results in higher levels of oxidative stress due to high-energy demands and an increase in oxygen utilization. Oxidative stress is a consequence of an imbalance between production of free radicals and their scavenging by the antioxidant systems (6,9).

Antioxidants have the capacity to neutralize free radicals and reactive oxygen species, and interfere with oxidation of lipids, nucleic acids and proteins. They can function at four levels: A) They prevent free radical formation.

B) They prevent or inhibit the activity of free radicals after they are formed.

C) They repair damages inflicted by the activity of free radicals.

D) They increase the excretion or absorption of damaged molecules (10).

There are two types of antioxidative agents in the body: [1] enzymatic, [2] non-enzymatic. The most important enzymatic antioxidants are superoxide dismutase (SOD), catalase and glutathione peroxidase (GPO) (11).

Due to the cumulative effect of antioxidants, it is advisable to measure the combined activity of all the antioxidants or the total antioxidant capacity (TAC) instead of measuring the activity of each agent separately (12,13). Total antioxidant capacity (TAC) has been defined as the moles of oxidants that are neutralized by one liter of solution; it is a biomarker used to measure the antioxidant potential of body fluids (14).

It has been reported that peroxidase activity, free-radical production and antioxidant defenses are disturbed in diabetes and pregnancy. Studies have shown that patients with type II diabetes mellitus exhibit severe oxidative stress (15), some studies demonstration enhanced oxidation products and lowered antioxidant capacity in patients suffering from GDM, indicating that oxidative stress contributes to the initiation and progression of GDM (15-19). In contrast, a different study showed that pregnant women with diabetes had higher salivary levels of TAC (20).

Saliva consists of water and organic and inorganic components, in addition to buffers, enzymes, growth factors, cytokines, immunoglobulins, mucins and other glycoproteins, as well as numerous defense systems such as the antioxidant defense system that are the first line of defense against oxidative stress (21,22).

Salivary components not only have a role in protecting oral tissues, but also they may reveal some systemic diseases. Therefore, salivary biomarkers might be used as tools for evaluating public health and for early diagnosis of some medical conditions (21,22). Saliva has several advantages over serum as a clinical tool because it is easy to collect and store saliva and it can be collected in sufficient amount for laboratory analyses. In addition, saliva can be collected as using non-invasive sampling techniques, provokes less anxiety and discomfort in patients and can be used for long-term evaluation of one particular condition (21-23).

In addition, it is much safer for researchers to collect saliva compared to blood tests because the latter is associated with the risk of exposure to hepatitis viruses or HIV. Therefore, the diagnosis of diseases with the use of saliva is much safer and cheaper than other methods (23,24). Pregnancy and diabetes increase free radical production and are associated with disturbances in antioxidant defenses. Considering the controversies in this area, especially with regard to the important role of antioxidants in the prevention and treatment of many medical conditions, this study was undertaken to investigate the total antioxidant capacity (TAC) of saliva in pregnant women whit gestational diabetes in comparison to non-diabetic pregnant women.

## Material and Methods

In this cross-sectional study, the study population consisted of patients referred to private clinics, the Department Oral Medicine, Tabriz Faculty of Dentistry and Al-Zahra Hospital in Tabriz during 2015-16. Being diabetic or non-diabetic and having any systemic disease and drug consumption in pregnant women was confirmed by an obstetrician using an approved checklist before sampling.

Screening for and diagnosis of GDM according to International Association of the Diabetes and Pregnancy Study Groups consensus: A 75-g OGTT (Oral Glucose Tolerance Test), with fasting plasma glucose measurement and at 1 and 2 hours, was carried out at 24-28 weeks of gestation in women not previously diagnosed with diabetes. The OGTT was performed in the morning after overnight fasting for at least 8 hours. The diagnosis of GDM was made when any of the following plasma glucose values were exceeded: Fasting: ≥92 mg/dL, 1-hour: ≥180 mg/dL, 2-hour: ≥153 mg/d (25).

A total of 31 women with a diagnosis of GDM and 59 non-diabetic pregnant women were included in the diabetic and control groups, respectively. All the subjects were matched for age, body mass index (BMI) and pregnancy with the control group.

Inclusion and exclusion criteria: The inclusion criteria consisted of a age range of 20-40 years, pregnant women with gestational diabetes, who were under treatment for diabetes, lack of any systemic diseases in the last three months, no past history of diabetes, not using any other drug not related with diabetes, no history of smoking, not using drugs that impair the balance of antioxidant defense system and intake of prescribed or over-the-counter antioxidants (such as vitamins and carotenes) in the past year. This information was obtained by studying medical records and performing clinical examinations.

Saliva collection method: Salivary samples were collected by spitting method, in which the patient was asked to collect her saliva in the mouth and then transfer it into a special sterile plastic Falcon tube. This was repeated every 60 seconds for 5-15 minutes. To collect unstimulated samples, each subject was asked to avoid eating or drinking or any other oral stimulation for 90 minutes prior to collecting the salivary samples. Using this method, almost 5 mL of saliva was collected. Saliva was collected for all the subjects between 8 and 9 am (fasting) to avoid diurnal changes. The collected salivary samples were immediately placed on ice and transported to the laboratory, followed by centrifugation at 800 g for 10 minutes at 4°C to separate squamous cells and debris. The samples were frozen at -80°C until preparation of all the samples was completed (26). When all samples were collected, total antioxidant capacity (TAC) was assayed.

Total antioxidant capacity (TAC) assay: Saliva TAC was measured with the use of a commercial kit following the manufacturer’s instructions (ZellBio GmbH, Germany) on the basis of the oxidation reduction colorimetric assay at a wavelength of 490 nm. TAC level was considered as the amount of antioxidant in the sample that was comparable to ascorbic acid action as a gold standard. This method can determine TAC with 0.1 mM sensitivity (100 µmol/L). The intra- and inter-assay variation coefficients have been reported to be less than 3.2% and 3.4%, respectively.

Ethical considerations: The study protocol conformed to the principles of the Declaration of Helsinki and was approved by the Medical Ethics Review Board of Tabriz University of Medical Sciences. Written informed consent was obtained from all the volunteers regarding confidentiality and anonymity of data collected. In addition, the details and aims of the study were disclosed. Patients were free to leave the study whenever the wished to.

Statistical analysis: Data were analyzed with descriptive statistics (means, standard deviations and frequency percentages) using SPSS 18. In order to compare the numeric values of the parameters, Mann-Whitney test was used because data were not distributed normally in the case and control groups. Kolmogorov-Smirnov test was used to show normal distribution of parameters. In this study statistical significance was set at *P*<0.05.

## Results

**Figure 1:**
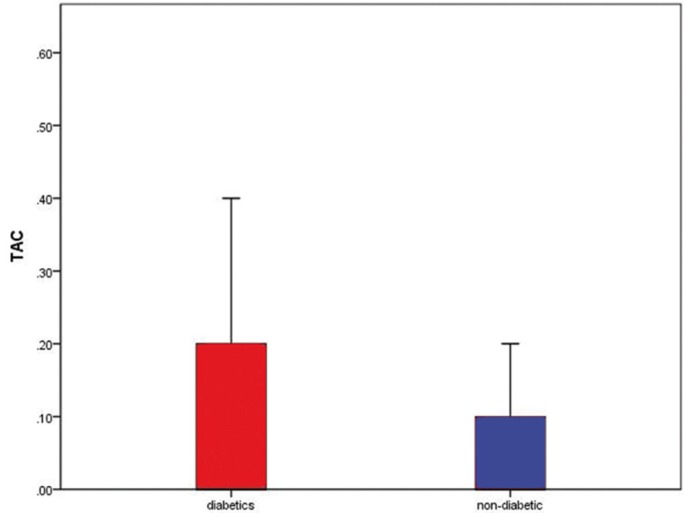
Comparison of total salivary antioxidant levels in pregnant women with and without diabetes.

Of 90 patients, 31 were women with gestational diabetes and 59 others were non-diabetic pregnant women. Average TAC level in the saliva of women with gestational diabetes was 0.10 ± 0.14, with 0.04 ± 0.11 in non-diabetic pregnant women. According to the data, average level of salivary TAC was higher in women with gestational diabetes (Fig. 1). Comparison of salivary TAC levels in women with gestational diabetes and non-diabetic pregnant women with nonparametric Mann-Whitney test (with respect to non-normal data) showed that this difference was statistically significant (*P*=0.024).

## Discussion

Gestational diabetes mellitus (GDM) is a common condition during pregnancy and is associated with some complications for the mother and fetus. Studies have shown that in societies with a higher rate of diabetes type II, gestational diabetes is more common, too, but the risk and development of the condition is variable (27). This condition is one of the most common complications of pregnancy, with a prevalence rate of approximately 7% (more than 2000 people per year) of all pregnancies in America (28).

Pregnancy is generally stressful (6,8) and is accompanied by a high-energy demand and an increase in oxygen consumption, which might give rise to increased oxidative stresses. Oxidative stresses occur when there is an imbalance between free radical production and the radical scavenging capacity of the antioxidant system. There is evidence that prooxidant, free radical production and antioxidant defenses are disturbed in diabetes and during pregnancy (6,9,29).

Recently, the saliva as an accessible clinical sample has been in the spotlight of the researchers’ attention due to it’s the possession of different enzymes and molecules and distinctive function for the diagnosis and treatment of various diseases and their complications (30,31).

The present study was undertaken to compare salivary TAC levels in women with GDM and pregnant women without diabetes and the results showed that salivary TAC levels were higher in pregnant women with diabetes. In a similar study in 2011, Surdacka *et al.* (20) evaluated 63 pregnant women with diabetes during the first semester of pregnancy and reported increased salivary TAC levels in such patients, consistent with the results of the present study. However, in contrast to the present study, there was no control group in that study.

Searches in PubMed, Google Scholar and EBSCO databases showed that the study above is the only study on the subject and other limited studies that are available have only evaluated salivary antioxidants separately and to date no study has compared salivary TAC in pregnant women with and without diabetes. However, it should be pointed out that evaluation of TAC is very important because salivary antioxidants have synergistic effects (12,13). In addition, the majority of studies have evaluated serum levels of antioxidants and only a few studies are available on salivary antioxidant levels. In a study by Shimada *et al.* (32) in 2016, the salivary melatonin levels were lower in pregnant women with diabetes compared to pregnant women without diabetes.

In a study by Grissa *et al.* (33) in 2007, total serum antioxidant defense capacity in diabetic mothers was lower compared to control subjects. In contrast, in another study in 2011 by Al-Shebly *et al.* (6) plasma levels of total antioxidants were significantly higher in diabetic women compared to the control group.

Such differences might be attributed to differences in sampling methods, differences in techniques used to examine the samples in the laboratory, differences in mean ages of the patients, differences in the time required to be affected by diabetes and differences in metabolic controls in patients.

The availability of only a limited number of studies and their contradictory results in relation to salivary and serum levels of antioxidants in pregnant women makes it difficult to reach a definite conclusion. However, under the limitations of the present study it might be concluded that during pregnancy there is an increase in oxidative stresses, resulting in an increase in total salivary antioxidant levels to counteract such stresses. Therefore, determining salivary antioxidative levels might be considered an alternative technique for the early diagnosis of diabetes. Therefore, it is suggested that longitudinal studies be carried out to evaluate variations in salivary levels of antioxidants in pregnant women from the early days of pregnancy until the last days of it in order to determine its validity for early diagnosis of GDM.

The limitations of the present study include impossibility of making the samples uniform in physiological, hormonal, nutritional and environmental terms, oral cavity status, status of disease control, medications and blood sugar levels. In addition, diabetes is a multifactorial disease and a variety of factors, including genetics, nutrition and environment, play a role in its etiology. In addition, the quantity and quality of salivary compositions might be influenced by genetics, amount and type of food, smoking, physical activity level, hormone structure, level of stress and the oral cavity conditions in relation to dental caries and periodontal diseases. Therefore, further studies are recommended with larger sample sizes and further consideration of confounding factors to achieve diagnostic and therapeutic protocols for diabetic patients.

Under the limitations of the present study it can be concluded that there is an increase in oxidative stresses during pregnancy, followed by an increase in the total levels of salivary antioxidants to counteract such stresses. Therefore, it is expected that determining the salivary antioxidant levels during pregnancy can be an alternative technique for the early diagnosis of diabetes.
